# Supplementary figures and images for: Target Of Rapamycin pathway in the white-rot fungus Phanerochaete chrysosporium
Source: PLoS One. 2020 Feb 20;15(2):e0224776. doi: 10.1371/journal.pone.0224776 (PMC7032718; doi:10.1371/journal.pone.0224776)

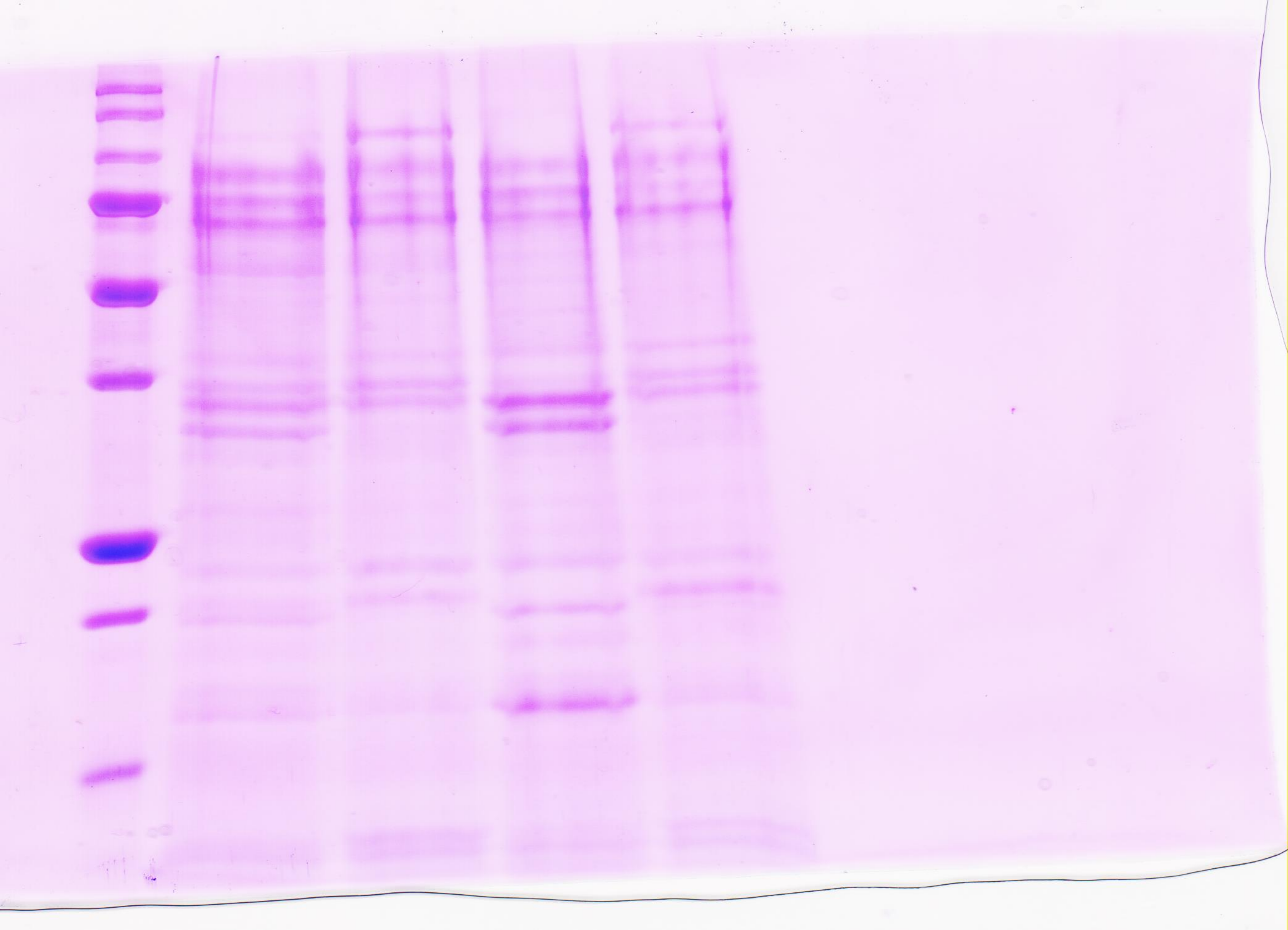

Supplement: S1 Raw Image — (PDF) [file pone.0224776.s003.pdf]
